# Inputs and outputs of insulin receptor

**DOI:** 10.1007/s13238-014-0030-7

**Published:** 2014-03-16

**Authors:** Yipeng Du, Taotao Wei

**Affiliations:** National Laboratory of Biomacromolecules, Institute of Biophysics, Chinese Academy of Sciences, Beijing, 100101 China

**Keywords:** insulin, insulin receptor, signaling

## Abstract

The insulin receptor (IR) is an important hub in insulin signaling and its activation is tightly regulated. Upon insulin stimulation, IR is activated through autophosphorylation, and consequently phosphorylates several insulin receptor substrate (IRS) proteins, including IRS1-6, Shc and Gab1. Certain adipokines have also been found to activate IR. On the contrary, PTP, Grb and SOCS proteins, which are responsible for the negative regulation of IR, are characterized as IR inhibitors. Additionally, many other proteins have been identified as IR substrates and participate in the insulin signaling pathway. To provide a more comprehensive understanding of the signals mediated through IR, we reviewed the upstream and downstream signal molecules of IR, summarized the positive and negative modulators of IR, and discussed the IR substrates and interacting adaptor proteins. We propose that the molecular events associated with IR should be integrated to obtain a better understanding of the insulin signaling pathway and diabetes.

## Introduction

Input and output are two basic concepts in the field of cellular signal transduction (Waltermann and Klipp, 2011). In general, the inputs of a signal transduction pathway are the upstream stimulation and inhibition signals, whereas the outputs are the downstream effects, such as the activation of substrates and interactions with other proteins. The classical insulin signaling pathway is initiated by the binding of insulin to the insulin receptor (IR) and the subsequent activation of insulin receptor substrate (IRS) proteins (Taniguchi et al., 2006). For the hub protein IR, the input is insulin, and the output is the phosphorylation of IRS proteins. However, the scenario is not as simple as this one-way signal transduction model. Recent progress in IR signaling indicated that insulin is not the only ligand for IR, and the phosphorylation of IRS proteins represents just one component of IR output. To provide a more complete understanding of the signals mediated through IR, we reviewed the inputs and outputs of IR in this study. We will first provide a brief description of the classical stimulus and substrates of IR and then mainly focus on the factors that positively and negatively regulate IR and the various IR substrates and interacting proteins.

## CLASSICAL INPUTS AND OUTPUTS OF IR

**Figure 1:**
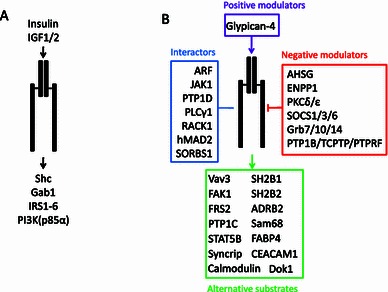
**The diagram illustrating the inputs and outputs of insulin receptor**. (A) Classical IR inputs and outputs. (B) Summary of proteins associating with IR which include positive modulator (purple), negative modulator (red), alternative substrates (green) and interactors (blue). Arrow: stimulation; Line: interaction; Arrow with flathead: inhibition

The binding of insulin to the α subunits of IR heterotetramer is the main input into the insulin signaling pathway. The IR then undergoes a conformational change in its intracellular β subunits that exposes its ATP-binding domain, which enables ATP binding and autophosphorylation. The output of IR activation is the phosphorylation of a group of IRS proteins (White and Kahn, 1994). IRS1 is the principal IRS protein and is phosphorylated at multiple tyrosine sites upon insulin stimulation (Sun et al., 1993). The tyrosine-phosphorylated IRS1 sites function by docking with SH2 domain-containing proteins and mediating signal transduction to various downstream factors. IRS2 is an alternative IR substrate that was discovered in IRS1-deficient mice (Patti et al., 1995). IRS1 and IRS2 are not functionally redundant, although they both activate many similar downstream pathways (Waters and Pessin, 1996; Hanke and Mann, 2009). IRS3 was first cloned in rat adipocytes and then in mouse. However, this homolog does not appear to exist in human cells (Lavan et al., 1997b; Sciacchitano and Taylor, 1997). IRS4 is the dominant IRS in HEK293 cells (Lavan et al., 1997a). Although IRS1 and IRS2 are present in the HEK293 cell line, they are not activated upon insulin stimulation (Fantin et al., 1998). These results indicate that the output of IR is tissue-specific. IRS5 and IRS6, also known as DOK4 and DOK5, are readily phosphorylated in CHO-IR cells (Cai et al., 2003). IR-phosphorylated IRS5 binds to a number of SH2 domain-containing proteins, that are distinct from those associated with IRS1. However, IR-phosphorylated IRS6 does not bind to any SH2 domain-containing proteins, which indicates that it may mediate a different branch of the insulin signaling pathway (Cai et al., 2003). Shc interacts with and is phosphorylated by IR. Phosphorylated Shc can dock with Grb2 and mediate signal transduction through the Ras-MAPK signaling pathway (Sasaoka and Kobayashi, 2000). Gab1 is another IRS protein that is mainly involved in the PI3K-Akt pathway (Lehr et al., 2000). The p85α subunit of PI3K can be directly phosphorylated by IR after its docking to IRS1/2 (Hayashi et al., 1993; Van Horn et al., 1994). Although the classical IR inputs and outputs are simple and clear, they are only part of the story (Fig. 1A).

In addition to the classical IRS proteins, many other proteins have been shown to associate with IR and to play a role in the insulin signaling pathway (Fig. 1B), which could be classified into four different types: (1) Positive modulators of IR (e.g., glypican-4 stimulates insulin function by binding to IR); (2) Negative modulators of IR [e.g., protein tyrosine phosphatases (PTPs), protein kinase C (PKC) isoforms, growth factor receptor-bound (Grb) and suppressors of cytokine signaling (SOCS) proteins]; (3) Alternative IR substrates involved in various biological processes; and (4) proteins that interact with IR, but are not its substrates. The following section will focus on these four types of proteins in an attempt to provide a complete description of the inputs and outputs of IR. The related proteins and references are summarized in Table 1.

**Table 1:** Proteins associated with the inputs and outputs of insulin receptor

Protein	Function	References
**Positive modulators**		
Glypican-4	Binds IR α subunits. Facilitates insulin-induced formational change of IR	Ussar et al. 2012
**Negative modulators**		
PTP1B	Major PTP involved in insulin signaling. Dephosphorylates IR tyrosine	Salmeen et al. 2000
TCPTP	Cooperates with PTP1B. Dephosphorylate IR tyrosine	Galic et al. 2003
PTPRF	Dephosphorylates IR tyrosine	Hashimoto et al. 1992
PKCδ	Predominant in muscle. Phosphorylates IR serine	Braiman et al. 2001
PKCε	Predominant in liver. Phosphorylates IR serine	Samuel et al. 2007
Grb7	Binds IR *in vitro* and *in vivo*	Kasus-Jacobi et al. 2000
Grb10	Binds IR. Blocks IRS1/2	Wick et al. 2003
Grb14	Binds IR. Blocks PTP1B	Nouaille et al. 2006
SOCS1	Binds IR. Interrupts IRS2	Ueki et al. 2004
SOCS3	Binds IR. Interrupts IRS1/2, STAT5B	Emanuelli et al. 2000
SOCS6	Binds IR. Interrupts IRS1	Mooney et al. 2001
ENPP1	Binds IR α subunits. Inhibits insulin-induced conformational change of IR	Maddux and Goldfine, 2000
AHSG	Binds IR β subunits extracellular region	Mathews et al. 2000
**Alternative substrates**		
ADRB2	Transmembrane protein. Phosphorylated by IR *in vitro* and *in vivo*	Baltensperger et al. 1996
Calmodulin	Calcium-dependent protein. Phosphorylated by IR *in vitro* and *in vivo*	Sacks et al. 1992
CEACAM1	Transmembrane protein. Phosphorylated by IR *in vitro* and *in vivo*	Poy et al. 2002a
Dok1	GAP-associated protein. Phosphorylated by IR *in vitro* and *in vivo*	Wick et al. 2001
FABP4	Fatty acid-binding protein. Phosphorylated by IR *in vitro*	Buelt et al. 1991
FAK1	Integrin signaling pathway. Phosphorylated by IR *in vitro*	Baron et al. 1998
FRS2	FGFR substrate. Phosphorylated by IR *in vitro*	Delahaye et al. 2000
PTP1C	Protein tyrosine phosphatase. Phosphorylated by IR *in vitro* and *in vivo*	Uchida et al. 1994
SH2B1	Interacts with IR and phosphorylated upon insulin stimulation *in vivo*	Kotani et al. 1998
SH2B2	Interacts with IR and phosphorylated upon insulin stimulation *in vivo*	Moodie et al. 1999
STAT5B	Transcriptional factor. Phosphorylated by IR *in vitro* and *in vivo*	Chen et al. 1997
SYNCRIP	RNA-binding protein. Phosphorylated by IR *in vitro*	Hresko and Mueckler, 2002
Sam68	RNA-binding protein. Phosphorylated by IR *in vitro* and *in vivo*	Sanchez-Margalet and Najib, 1999
Vav3	Interacts with and phosphorylated by IR activation *in vivo*	Zeng et al. 2000
**Proteins interacting with IR, but are not its substrates (Interactors)**		
ARF	Interacts with activated IR. PLD regulation	Shome et al. 1997
hMAD2	Interacts with inactivated IR	O’Neill et al. 1997
JAK1	Interacts with activated IR. Enhances IRS1 activation	Gual et al. 1998
PLCγ1	Interacts with activated IR. Enhances MAPK activation	Kwon et al. 2003
PTP1D	Interacts with activated IR. Enhances IRS1 activation	Kharitonenkov et al. 1995
RACK1	Interacts with activated IR. Facilitates STAT3 activation	Zhang et al. 2006
SORBS1	Interacts with inactivated IR	Lin et al. 2001

## POSITIVE MODULATORS OF IR

Insulin, IGF1 and IGF2 are traditional IR ligands. Some adipokines have also been found to interact with the IR α subunits and to enhance insulin sensitivity. Glypican-4, which is released from adipose tissue into the circulation, is a potential IR ligand. It binds to IR at regions different from that of insulin (Ussar et al., 2012). The depletion of glypican-4 reduces insulin signaling, whereas the overexpression of wild-type glypican-4 enhances the insulin-mediated phosphorylation of ERK and AKT (Ussar et al., 2012), indicating its potential role as a target for the treatment of insulin resistance (Mitchell, 2012). Visfatin is related to the insulin signaling pathway, but its role in the stimulation of IR remains controversial (Adeghate, 2008).

## NEGATIVE MODULATORS OF IR

The activity of IR is negatively regulated by several mechanisms. PTPs can dephosphorylate IR at autophosphorylated tyrosines and thus inactivate IR. Members of the PKC family phosphorylate serines near autophosphorylated tyrosine sites to disrupt the docking of SH2 domain-containing proteins. Proteins in the Grb and SCOS families bind directly to IR and block its interaction with downstream factors. In addition, other proteins, such as NEPP1 and AHSG, inhibit IR by interacting with its extracellular domain. This indicates that a variety of negative modulators of IR cooperate to inactivate IR. We here summarized the important negative modulators of IR.

### PTPs (PTP1B, PTP1C, TCPTP and PTPRF)

PTPs are encoded by approximately 100 genes in humans (Alonso et al., 2004; Tonks, 2006). Classical PTPs dephosphorylate tyrosine phosphorylation to attenuate the function of many receptor tyrosine kinases (Andersen et al., 2001; Andersen et al., 2004). At least three PTPs proteins have been found to be involved in the negative regulation of IR by dephosphorylation. PTP1B is the best-known and most-studied PTP and regulates IR activity via dephosphorylation. It can be recruited to multiply tyrosine phosphorylation sites on the IR through its SH2 domain upon insulin stimulation and IR autophosphorylation (Seely et al., 1996). The tyrosines of PTP1B are then phosphorylated by IR, and this step greatly increases its dephosphorylation activity (Dadke et al., 2001). Phosphorylated PTP1B can dephosphorylate IR and inhibit its kinase activity (Salmeen et al., 2000). This typical negative feedback is widely present in biological processes. In addition, phosphorylated PTP1B can dephosphorylate itself to balance its catalytic activity. PTP1B has been recognized as a potential target for the enhancement of insulin sensitization through a series of functional studies (Delibegovic et al., 2007; Picardi et al., 2008; Delibegovic et al., 2009; Ma et al., 2011). Similar to PTP1B, TCPTP uses autophosphorylated IR as a direct substrate both *in vivo* and *in vitro* (Lammers et al., 1993; Galic et al., 2003). Although both PTP1B and TCPTP inhibit the activity of IR by dephosphorylation, the catalytic tyrosine sites might be different. PTP1B prefers to tandem phosphorylate tyrosine, whereas TCPTP mainly catalyzes a single phosphorylated tyrosine (Galic et al., 2005). PTPRF, which is a transmembrane PTP, also interacts with and dephosphorylates IR *in vitro* and *in vivo* (Hashimoto et al., 1992; Ahmad and Goldstein, 1997). It can be deduced that PTP1B, TCPTP and PTPRF crosstalk with each other to negatively regulate IR. In summary, three PTPs can act as negative inputs to IR via tyrosine dephosphorylation under conditions of insulin stimulation.

### PKCs (PKCδ and PKCε)

The PKC family consists of three distinct groups, namely, the classical, novel and atypical groups. In contrast to PTPs, which dephosphorylate proteins, PKCs function by phosphorylating serines or threonines. Several members in the PKC family have been shown to be involved in negatively regulating IR activity. *In vitro*-purified PKC can also phosphorylate IR and lower its tyrosine kinase activity (Bollag et al., 1986). *In vivo* studies using insulin-resistant human skeletal muscle suggest that PKCδ is recruited to IR and reduces its activity via serine phosphorylation (Itani et al., 2000; Braiman et al., 2001; Rosenzweig et al., 2004). In insulin-resistant liver, PKCε is the predominant activated PKC. PKCε inhibits insulin signaling by binding to IR and reducing its tyrosine kinase activity in hepatic steatosis (Samuel et al., 2007; Jornayvaz et al., 2011; Jornayvaz and Shulman, 2012). These studies indicate that PKC isoforms regulate IR activity in a tissue-specific manner. In addition, PKC can inhibit the insulin signaling pathway by phosphorylating other proteins in the insulin signaling pathway. For example, PKCθ prefers IRS1 for phosphorylation (Griffin et al., 1999; Yu et al., 2002; Li et al., 2004). In summary, at least two PKCs act as negative inputs to IR via serine phosphorylation.

### Grb proteins (Grb10, Grb14 and Grb7)

Unlike PTPs and PKC, which inhibit IR by covalently modification, Grb proteins reduce the activity of IR through direct interaction. The autophosphorylated tyrosine sites on IR not only dock IRS but also SH2 domain-containing proteins, which are not phosphorylated or activated by IR. These types of proteins compete with IRS for IR binding and then serve to inhibit IR activity. Grb10 was first found to be a high affinity interacting protein with IR *in vitro* (Liu and Roth, 1995). Further studies demonstrated that the Grb10 SH-2 domain and IR carboxyl catalytic active loop are required for this interaction (Hansen et al., 1996). Grb10 binds to the same domain as IRS1 on IR. This binding blocks the IR-mediated phosphorylation of IRS1 and disrupts the IRS1-PI3K signaling pathway (Wick et al., 2003). The biological role of Grb10 in promoting insulin signaling has been proven using a mouse model (Smith et al., 2007; Wang et al., 2007). Similar to Grb10, Grb14 binds to IR and blocks its autophosphorylation in a site-specific manner (Nouaille et al., 2006). The phenotypes of mice deficient in Grb14 and Grb10 differ, which indicates the non-redundant functions of these two proteins (Holt and Siddle, 2005). Grb7 is an additional Grb protein that participates in IR regulation. It binds to activated IR both *in vitro* and *in vivo* and may function in the same manner as Grb14 and Grb10 (Kasus-Jacobi et al., 2000). In summary, three Grb proteins can inhibit IR activity by directly blocking downstream signaling.

### SOCS proteins (SOCS1, SOCS3 and SOCS6)

Initially identified as cytokine signaling inhibitors, SOCS proteins participate in various signal transduction pathways, including the insulin signaling pathway. Similar to Grb proteins, SOCS proteins directly interact with IR and block downstream signal transduction. For example, SOCS3 attenuates the IR-STAT5B signal branch by competing with STAT5B for binding to IR at phosphorylated tyrosine 960 (Emanuelli et al., 2000). SOCS3 also interrupts the IRS1 and IRS2 signal branch with the same mechanism (Ueki et al., 2004). Moreover, SOCS3 overexpression in cultured cells inhibits IR autophosphorylation. This mechanism is most likely mediated by crosstalk between IR regulators (Senn et al., 2003). In addition, SOCS3 is induced by STAT5B upon insulin stimulation (Emanuelli et al., 2000; Sadowski et al., 2001). This constitutes another negative feedback loop in the insulin signaling pathway. SOCS1 binds to IR on sites different from that of SOCS3, although they are both IR inhibitors (Mooney et al., 2001; Le et al., 2002; Ueki et al., 2004). SOCS6 binds to both IR and IRS4, which indicates that it inhibits the insulin signaling pathway by targeting multiple proteins (Mooney et al., 2001; Krebs et al., 2002). SOCS proteins can be induced by inflammation, which partially explains the role of inflammation in insulin resistance (Tanti et al., 2012; Suchy et al., 2013). We concluded that at least three SOCS proteins provide negative inputs to IR.

### ENPP1

ENPP1 (ectonucleotide pyrophosphatase/phosphodiesterase family member 1) is a transmembrane protein with alkaline phosphodiesterase and nucleotide pyrophosphatase activity. However, its inhibition of IR autophosphorylation is independent of its enzymatic activity (Grupe et al., 1995). In contrast to Grb and SOCS proteins, which inhibit IR activity by interacting with its β subunits, ENPP1 directly binds to the IR α subunits (Maddux and Goldfine, 2000). ENPP1 does not appear to affect insulin binding, but inhibits the insulin-induced conformational change of the IR α subunits (Maddux and Goldfine, 2000). The ENPP1 K173Q polymorphism (previously described as K121Q) has been associated with insulin resistance (Costanzo et al., 2001; McAteer et al., 2008; Moore et al., 2009) because the K173Q polymorphism tightly binds to IR and effectively inhibits IR. There are seven members in the ENPP family (Masse et al., 2010). Given the similarity between ENPP1 and the other ENPPs, particularly ENPP2 (Kato et al., 2012), it is possible that these other ENPPs may participate in the insulin signaling pathway. Furthermore, the up-regulation of ENPP2 has been associated with insulin resistance in a diabetic mouse model (Boucher et al., 2005). Thus, it can be concluded that ENPP1 inhibits IR by blocking the conformational change of IR upon insulin binding.

### AHSG

AHSG (alpha-2-Heremans-Schmid glycoprotein) inhibits the autophosphorylation of IR by interacting with the extracellular region of its β subunits (Auberger et al., 1989; Srinivas et al., 1993; Mathews et al., 2000). The mechanism of this inhibition may be similar to that of ENPP1. AHSG is secreted by the liver and is released into the circulation. The blood levels of AHSG have been correlated with insulin resistance (Kalabay et al., 2002; Goustin and Abou-Samra, 2011). Thus, it is becoming clear that AHSG is another negative input of IR.

## ALTERNATIVE SUBSTRATES OF IR

IR substrates are not restricted to the classical ones mentioned above. A variety of proteins have been found to be phosphorylated by activated IR. We categorized these proteins into different IR outputs and review their roles in the insulin signaling pathway in this section.

### ADRB2

ADRB2 (beta-2-adrenergic receptor) is a member of the G-protein coupled receptor superfamily and can be phosphorylated by IR both *in vivo* and *in vitro* (Baltensperger et al., 1996). The phosphorylated tyrosine site on ADRB2 can recruit Grb2 and other proteins to promote the internalization of ADRB2 (Karoor et al., 1998). This indicates that the insulin signaling pathway may crosstalk with the GPCR (G-protein coupled receptor) signaling pathway.

### Calmodulin

Calmodulin is a multifunctional calcium-dependent messenger protein that can be phosphorylated by different types of kinases, including IR (Laurino et al., 1988; Sacks and McDonald, 1988; Wong et al., 1988; Sacks et al., 1989; Sacks and McDonald, 1989; Sacks et al., 1992). The phosphorylation of calmodulin has an effect on its intrinsic enzyme property and on its downstream interacting proteins (Benaim and Villalobo, 2002). IR phosphorylates calmodulin at two major tyrosine sites, which attenuates its biological activity (Saville and Houslay, 1994; Williams et al., 1994; Sacks et al., 1995; Joyal et al., 1996). However, the precise role of this phosphorylation related to insulin signaling is unclear. Calmodulin may serve as a node for the crosstalk between the insulin signaling pathway and other signaling pathways.

### CEACAM1

CEACAM1 (carcinoembryonic antigen-related cell adhesion molecule 1) is another transmembrane substrate of IR. IR phosphorylates CEACAM1 on its intracellular domain to initiate IR internalization (Formisano et al., 1995). CEACAM1-mediated IR internalization and degradation is important for insulin clearance in the liver (Poy et al., 2002b). The tyrosine-phosphorylated sites on CEACAM1 also compete with IR for Shc binding, thus attenuating IR signaling transduction to the MARK pathway (Poy et al., 2002a).

### Dok1

Dok1 (docking protein 1) is phosphorylated by IR at specific tyrosine sites. Phosphorylated Dok1 enhances its binding to GAP, which is an inhibitor of RAS (Wick et al., 2001). Thus, RAS is dually regulated by the insulin signaling pathway: it is activated by Grb2-SOS and inactivated by Dok1-GAP. Dok2 and Dok3 do not appear to be related to the insulin signaling pathway, but these proteins interact with GAP and may play an important role in other pathways (Di Cristofano et al., 1998; Lemay et al., 2000).

### FABP4

FABP4 (fatty acid-binding protein 4) is mainly expressed in adipocytes, which can be phosphorylated by the purified IR β subunits *in vitro* (Buelt et al., 1991). In addition, results obtained from FABP4-deficient mice indicate that this protein may serve as a bridge linking obesity to insulin resistance (Hotamisligil et al., 1996). However, it remains unclear whether FABP4 is a substrate of IR *in vivo* and whether it participates in the insulin signaling pathway.

### FAK1

FAK1 (focal adhesion kinase 1) is a cytosolic tyrosine kinase involved in integrin signaling. IR promotes FAK1 phosphorylation in suspended cells (Baron et al., 1998). In contrast, IR stimulates the dephosphorylation of FAK1 in attached cells (Pillay et al., 1995). The biological role of IR-phosphorylated FAK1 remains to be elucidated. The dual role of IR in FAK1 regulation may be related to the integrin-mediated signaling pathway.

### FRS2

FRS2 (fibroblast growth factor receptor substrate 2) was originally found to be an adapter protein that links activated FGR receptors to the downstream signaling pathway (Xu et al., 1998). It has also been revealed to be a direct substrate of IR *in vitro* and becomes tyrosine phosphorylated upon insulin stimulation *in vivo* (Delahaye et al., 2000).

### PTP1C

PTP1C is able to bind to autophosphorylated IR and is phosphorylated by activated IR on its tyrosine residues (Uchida et al., 1994). In addition, the phosphatase activity of phosphorylated PTP1C increases. However, there is no evidence demonstrating that it can directly dephosphorylate IR, although PTP1C-deficient mice exhibit increased glucose tolerance and insulin sensitivity (Dubois et al., 2006).

### SH2B1/2

SH2B1 and 2 are SH2 domain-containing proteins that can be tyrosine phosphorylated upon insulin stimulation (Yokouchi et al., 1997; Kotani et al., 1998; Wang and Riedel, 1998; Moodie et al., 1999). The insulin-induced phosphorylation of SH2B1/2 is potentially mediated by IR. Similar to Grb2 and Shc, phosphorylated SH2B1 and 2 may serve as a docking site for downstream factors. For example, phosphorylated SH2B2 can dock c-Cbl to IR and promote IR ubiquitination and internalization (Ahmed et al., 2000). In addition, SH2B 1 and 2 serve as an adaptor and substrates for other tyrosine kinase receptors, such as receptors of PDGF (platelet-derived growth factor), NGF (nerve growth factor), and FGF (fibroblast growth factor) (Rui and Carter-Su, 1998; Kong et al., 2002; Wang et al., 2004).

### STAT5B

STAT5B (signal transducer and activator of transcription 5B) has been demonstrated to be a direct substrate for IR both *in vitro* and *in vivo* (Chen et al., 1997; Sawka-Verhelle et al., 1997; Storz et al., 1999; Sawka-Verhelle et al., 2000). Phosphorylated STAT5B acts as a transcription factor and activates a series of target genes, including glucokinase and SOCS proteins (Sawka-Verhelle et al., 2000; Sadowski et al., 2001). Insulin-induced gene expression events may be partially mediated by STAT5B.

### SYNCRIP and Sam68

IR substrates are not restricted to cytoplasmic enzymes and transcriptional factors. IR also phosphorylates RNA-binding proteins. For instance, the RNA-binding protein SYNCRIP (synaptotagmin-binding cytoplasmic RNA-interacting protein) is phosphorylated by IR *in vitro* (Hresko and Mueckler, 2000, 2002). Moreover, this phosphorylation can be disrupted by RNA binding. The RNA-binding protein Sam68, which can be induced by insulin, is phosphorylated by IR both *in vitro* and *in vivo* (Sanchez-Margalet and Najib, 1999; Sanchez-Margalet et al., 2003). Tyrosine-phosphorylated Sam68 (the 68 kDa Src substrate associated during mitosis) can dock with p85 PI3K and GAP proteins (Sanchez-Margalet and Najib, 2001). The RNA-binding activity of Sam68 is also affected by tyrosine phosphorylation (Wang et al., 1995), indicating a role of the insulin signaling pathway in RNA metabolism.

### Vav3

Vav3 is a member of the guanine nucleotide exchange factor family, which activates multiple pathways. Vav3 interacts with and is phosphorylated by IR when overexpressed in 293T cells (Zeng et al., 2000). IR-phosphorylated Vav3 promotes Rac-1 activation and actin cytoskeletal rearrangement and modulates the formation of cell membrane ruffles (Zeng et al., 2000).

## PROTEINS INTERACT WITH IR BUT ARE NOT ITS SUBSTRATES

IR does not always provide an output signal via catalysis. Under certain conditions, signals from IR are transmitted by changes in its interaction status with its binding partners. For example, inactivated IR interacts with hMAD2 (human homolog of yeast MAD2) with high affinity. This interaction decreases upon IR activation (O’Neill et al., 1997). Similarly, SORBS1 (sorbin and SH3 domain-containing 1) dissociates from IR and binds to c-Abl upon insulin stimulation (Lin et al., 2001). Although the biological function of these types of proteins in the insulin signaling pathway remains unclear, they enable signal transduction through IR.

In cells overexpressing IR, JAK1 (Janus kinase 1) has been observed to interact with IR (Gual et al., 1998). In addition, the phosphorylation of both proteins is necessary for their interaction. The binding of JAK1 to IR may facilitate its catalytic activity to IRS1. RACK1 (receptor for activated C kinase 1) interacts with both IR and STAT3 *in vitro*. In addition, RACK1 mediates IR-induced STAT3 activation *in vivo* (Zhang et al., 2006). These results indicate that IR transmits signals to STAT3 via RACK1. ARF (ADP-ribosylation factor) has been coimmunoprecipitated with activated IR and serves as an adaptor to IR-mediated PLD regulation (Shome et al., 1997). In addition, PTP1D (protein tyrosine phosphatase 1D), which is a member of the PTP family, can bind to both IR and IRS1 and enhance the docking of IRS1 to IR (Kharitonenkov et al., 1995). PLCγ1 (phospholipase C gamma 1) can interact with activated IR in a SH2 domain-independent manner, which may be mediated by conformational changes of IR (Kwon et al., 2003). The binding to PLCγ1 leads to phosphorylation, which plays a role in signal transduction to MAPK. Thus, the IR-mediated signals can be transmitted by interacting proteins independent of its catalytic activity.

## Conclusions

After four decades of extensive investigation, a growing body of knowledge on IR has been accumulated. In this review, we summarized the signals input to or output from IR (Fig. 1). Although each signal branch through IR is clear and understandable, the integration of all of the signal branches to obtain a full understanding of insulin signaling remains challenging. To fully understand the IR signaling cascades, it is not sufficient to study IR-interacting proteins or signal branches one-by-one; all related proteins and pathways must be integrated. The typical cellular response to insulin stimulation is the phosphorylation of classical IRS and IRS-mediated macromolecular complex docking. However, we should consider other independently reported docking proteins, such as Dok1, SH2B1 and SH2B2 to the macromolecular docking process. In parallel with the docking event is the direct activation of a group of proteins, including calmodulin, STAT5B and SYNCRIP. We should also integrate the negative regulation mechanisms with the activation event triggered by IR. The represented members of negative regulators are PTPs, PKCs, Grb and SOCS proteins. In addition, insulin may be dissociated or internalized and degraded via a CEACAM-mediated mechanism. Add there should be other IR related proteins and events which remain to be elucidated. Only if the input and output signals are integrated into one story, we may eventually obtain a full understanding of IR signaling.

## Abbreviations

CEACAM1: carcinoembryonic antigen-related cell adhesion molecule 1
Dok1: docking protein 1
FABP4: fatty acid-binding protein 4
FRS2: fibroblast growth factor receptor substrate 2
Grb: growth factor receptor-bound
IR: insulin receptor
IRS: insulin receptor substrate
JAK1: Janus kinase 1
PKC: protein kinase C
PTP: protein tyrosine phosphatase
RACK1: receptor for activated C kinase 1
Sam68: the 68 kDa Src substrate associated during mitosis
SOCS: suppressors of cytokine signaling
SORBS1: sorbin and SH3 domain-containing 1
STAT5B: signal transducer and activator of transcription 5B
SYNCRIP: synaptotagmin-binding cytoplasmic RNA-interacting protein
